# The Mothers, Infants, and Lactation Quality (MILQ) Study: A Multi-Center Collaboration

**DOI:** 10.1093/cdn/nzab116

**Published:** 2021-09-20

**Authors:** Lindsay H Allen, Daniela Hampel, Setareh Shahab-Ferdows, Maria Andersson, Erica Barros, Andrew M Doel, Kamilla Gehrt Eriksen, Sophie Hilario Christensen, Munirul Islam, Gilberto Kac, Farhana Khanam Keya, Kim F Michaelsen, Daniela de Barros Mucci, Fanta Njie, Janet M Peerson, Sophie E Moore

**Affiliations:** USDA, Agricultural Research Service (ARS) Western Human Nutrition Research Center, Davis, CA, USA; Department of Nutrition, University of California, Davis, CA, USA; USDA, Agricultural Research Service (ARS) Western Human Nutrition Research Center, Davis, CA, USA; Department of Nutrition, University of California, Davis, CA, USA; USDA, Agricultural Research Service (ARS) Western Human Nutrition Research Center, Davis, CA, USA; Department of Nutrition, University of California, Davis, CA, USA; Nutrition Research Unit, University Children's Hospital Zurich, Zurich, Switzerland; Federal University of Rio de Janeiro, Rio de Janeiro, Brazil; King's College London, London, United Kingdom; Department of Nutrition, Exercise, and Sports, University of Copenhagen, Copenhagen, Denmark; Department of Nutrition, Exercise, and Sports, University of Copenhagen, Copenhagen, Denmark; Nutrition and Clinical Services Division, International Centre for Diarrhoeal Disease Research, Bangladesh (icddr, b), Dhaka, Bangladesh; Federal University of Rio de Janeiro, Rio de Janeiro, Brazil; Nutrition and Clinical Services Division, International Centre for Diarrhoeal Disease Research, Bangladesh (icddr, b), Dhaka, Bangladesh; Department of Nutrition, Exercise, and Sports, University of Copenhagen, Copenhagen, Denmark; Federal University of Rio de Janeiro, Rio de Janeiro, Brazil; Medical Research Council Unit The Gambia at London School of Hygiene & Tropical Medicine, Fajara, The Gambia, West Africa; USDA, Agricultural Research Service (ARS) Western Human Nutrition Research Center, Davis, CA, USA; Federal University of Rio de Janeiro, Rio de Janeiro, Brazil; Medical Research Council Unit The Gambia at London School of Hygiene & Tropical Medicine, Fajara, The Gambia, West Africa

**Keywords:** human milk, macronutrients, micronutrients, composition, reference values

## Abstract

Little valid information is available on human milk nutrient concentrations, especially for micronutrients (MNs), and there are no valid reference values (RVs) across lactation. In this multi-center collaborative study, RVs will be established for human milk nutrients across the first 8.5 mo postpartum. Well-nourished, unsupplemented women in Bangladesh, Brazil, Denmark, and The Gambia (*n* = 250/site) were recruited during the third trimester of pregnancy. Milk, blood, saliva, urine, and stool samples from mothers and their infants are collected identically at 3 visits (1–3.49, 3.5–5.99, 6.0–8.49 mo postpartum). Milk analyses include macronutrients, selected vitamins, trace elements and minerals, iodine, metabolomics, amino acids, human milk oligosaccharides, and bioactive peptides. We measure milk volume; maternal and infant diets, anthropometry, and morbidity; infant development, maternal genome, and the infant and maternal microbiome. RVs will be constructed based on methods for the WHO Child Growth Standards and the Intergrowth-21st Project. This trial was registered at clinical trials.gov as NCT03254329.

## Teaser

This article describes the protocol for an international study of human milk composition that will enable the construction of reference values for milk nutrient concentrations during lactation.

## Introduction

For the first 6 mo of life, the WHO recommends exclusive breastfeeding (EBF) (1). However, human milk is not only essential for optimal infant health and development during the first 6 mo, but it can also be an important source of nutrients and other factors for the following ≈18 mo (2), yet we lack valid information on its nutrient content (3, 4), especially for micronutrients (MNs). Many human milk MN concentrations are much lower where women consume poor diets (5, 6), including MNs of major public health importance, such as vitamin A, thiamin (B-1), B-12, and iodine (7–9). These low milk concentrations caused by maternal deficiency and/or low intake have documented adverse effects on infant health and development (10), e.g. growth faltering and developmental delays have been linked to low milk concentrations in conjunction with severe maternal-infant deficiencies of vitamin B-12, B-1, B-6, D, iodine, and choline (9, 11).

In 2006, the new WHO International Child Growth Standards exposed that growth faltering starts shortly after birth with a much higher prevalence during the first 6 mo than previously assumed (12). During the same timeframe for infancy, the high prevalence of MN deficiency has been reported in exclusively or predominantly breastfed infants (6), e.g. thiamin status in Cambodia, which expands to mothers and milk, and low status of several MNs in Bangladesh (13, 14), suggesting poor milk quality could contribute to the growth faltering and MN deficiency.

The last and only global data on milk composition with a focus on MNs were collected in the 1985 WHO Collaborative Study on Breast-Feeding, over 30 y ago, with limited data collection (15). Our recent literature review on human milk revealed the general lack of a systematic sample collection and of appropriate consideration of factors affecting milk nutrient concentrations, e.g. stage of lactation, maternal nutritional status and diet, or smoking and alcohol use, among others (4, 16, 17). Moreover, inadequate methods for analyzing nutrients in the human milk matrix and lack of reported validation data added to the unreliability of many past reports (3, 18). Therefore, the literature on human milk composition is very difficult to interpret and practically impossible to use for the purposes for which such information is needed.

Due to all these limitations, there are no adequately established reference values (RVs) for nutrient concentrations in milk, and recommended nutrient intakes of infants, young children, and lactating women are based on many unvalidated and incorrect estimates of milk MN concentrations. Given the lack of RVs there is no benchmark against which to evaluate human milk quality in different populations, or the possible need for or effects of nutrient interventions on human milk MNs.

The primary study outcome and analysis objective of the Mothers, Infants, and Lactation Quality (MILQ) Study is the construction of reference ranges for values of vitamin and mineral concentrations in human milk from well-nourished mothers and their healthy infants. The reference range curves, between the 2.5 and 97.5 percentiles, will be constructed between 1 and 8.5 mo by combining data from the 4 study sites, using nutrient and volume data from milk samples obtained from each mother during 3 visits postpartum. This article describes the methodologies and protocols implemented for conducting the MILQ Study.

## Methods

### Study design and settings

The MILQ Study is a multi-center, collaborative project with data and sample collection in 4 countries, Bangladesh, Brazil, Denmark, and The Gambia. Samples and data (e.g. anthropometry, questionnaires) are collected from 250 mother-infant dyads per country site (*n *= 1000 mothers and 1000 infants) in a systematic, identical way across sites for colostrum (1–2 d postpartum), and at 3 subsequent time points: 1.0–3.49 mo (visit 2), 3.5–5.99 mo (visit 3), and 6.0–8.5 mo (visit 4) postpartum (*n* = 1000 dyads). Within each time window the collection of samples is randomized so that some samples and data are available for almost every day between 1 and 8.5 mo postpartum, enabling smoothing of reference curves. Visits 2 through 4 are divided into 3- or 4-wk time periods. If a participant's first visit is randomly assigned to the first week of a time period, then their second and third visits are also scheduled in the first week of the time block. To evaluate the effect of intraperson variability on outcome variables, a subset of 50 women in Bangladesh, Brazil, and The Gambia are providing a second milk sample on visits 2 and 3. Details about each study location and analytical site are provided in **Tables 1** and **2**.

**TABLE 1:** Study settings, and participant information in the Mothers, Infants, and Lactation Quality (MILQ) Study

	Bangladesh	Brazil	Denmark	The Gambia
Location	Mirpur (periurban area of Dhaka)	Madureira and São Cristóvão (Rio de Janeiro)	Copenhagen Rigshospitalet	Bakau and Fajara urban Gambia near Banjul
Average income, USD/mo	245	456	3120	247
Literacy, %	69	93	100	>65
Maternity leave, mo	6^1^	4–6	9–12	6
EBF, %				
2 mo	_	50	71	70
4 mo	_	_	60	_
6 mo	55	_	_	47
Infant stunting at 6 mo, %	18.6	4.8	<1	9
Usual diet	Rice	Rice and beans	Typical Western diet	Rice and maize
	Fruit and vegetables	Fruit and vegetables	Animal source foods, dairy	Fruit and vegetables
	Some animal source foods	Animal source foods, dairy		Groundnuts
		Pasta, high-processed foods		Some animal source foods
MMN supplementation	No, unless prescribed		Some, but not in this study	No
*Government mandates or recommendations*				
Perinatal	_	_	Iron and folic acid	_
Pregnancy	_	Iron and folic acid	Vitamin D	Iron and folic acid
	_	_	Calcium (low cow milk intake)	_
Fortification programs	No	Iron and folic acid (flour)	Iodine (salt)	No
Facilities	icddr, b, Dhaka	Maternity Hospital Herculano	Hvidovre Hospital	SOS Mother and Child Clinic,
		Pinheiro	Herlev Hospital	Bakoteh
		Municipal Center of Health	Rigshospitalet	
		Ernesto Zeferino Tibau Junir		
Type of informed consent	Written consent by study participant^2^			
Sample storage and use	Sample use is explained and consented to in the informed consent document			
	Samples are available for 5 y	Samples are available for 10 y	Samples are available for 5 y	
	Samples may be used for other projects but may require additional consent			Samples may be used for other projects
Deidentification and data security	All data is collected by study ID only into a secure database, at which point it cannot be linked back to the study participants			
	Separate, password-protected participant log is maintained by research team			
Compensation	Free treatment of any conditions by study physician	Travel costs	Breastfeeding counseling by phone, if desired	Travel costs
		Breastfeeding counseling	A small gift for the infant upon study completion	
Follow-up	If needed, referrals are provided to government specialized hospitals for further management	If needed, determined by study nurse, referrals are provided to specialized care facilities for further management^3^	A medically trained person is available for participants experiencing any illness, or if any abnormal test results	If needed, determined by study nurse, referrals are provided to MRCG@LSHTM, Fajara, clinic for further management

1 For government employees, varies for private entities.

2 If women are unable to read the consent form, it is read to them in their mother tongue and full informed consent is confirmed by *1*) the research staff (Bangladesh, Brazil), and *2*) by a literate, independent witness (The Gambia).

3 Issues related *1*) to illness: public Basic Health Unit, *2*) to breastfeeding issues: human milk bank at study site, *3*) to depression: Psychiatric Institute at the University. EBF, exclusive breastfeeding; icddr, b, International Centre for Diarrhoeal Disease Research, Bangladesh; MRCG@LSHTM, The MRC Unit The Gambia at the London School of Hygiene and Tropical Medicine; MMN, multiple micronutrients; PI, principal investigator; USD, US dollars.

**TABLE 2:** Analytical sites of the Mothers, Infant, and Lactation Quality (MILQ) Study

Site	Analyses
USDA, ARS-WHRNC^1^	Macro- and micronutrients, and metabolomics
	Selected micronutrients in colostrum based on available volume
	Microbial community of infant stool by 16s rRNA sequencing
	Shotgun metagenomics sequencing of infant stool based on 16s rRNA
	Screening, DNA quality/quantity, and availability of other data
	Milk cell mRNA transcriptome
	Maternal genetic and epigenetic signatures (buffy coat)
UC Davis, USA^2^	Human milk oligosaccharides and proteomics
ETH Zürich^3^	Iodine and iodine status biomarkers
St. John's Medical College^4^	Milk volume by D_2_O saliva analyses
University of Cambridge, UK^5^	Vitamin D in human milk

1 USDA, Agricultural Research Service-Western Human Nutrition Research Center, Davis, CA, USA.

2 University of California, Davis, CA, USA.

3 Eidgenössische Technische Hochschule, Zürich, Switzerland.

4 Bengaluru, India.

5 Nutritional Biomarker Laboratory, MRC Epidemiology Unit, University of Cambridge, Cambridge, UK.

D_2_O, deuterated water.

### Inclusion criteria

Participant's inclusion and exclusion criteria vary across the study periods but are focused mainly on the nutritional status and health of the mother and infant, and the requirement for EBF during the first 3.5 mo postpartum. Detailed information about the inclusion and exclusion criteria are provided in **Table 3**.

**TABLE 3:** Inclusion and exclusion criteria in the Mothers, Infants, and Lactation Quality (MILQ) Study

	Bangladesh	Brazil	Denmark	The Gambia
Recruitment	Directly by local research team	Invitation letter to meet	Directly by local research team	
Enrollment	After delivery		During pregnancy	
*Mothers inclusion criteria*				
Age, y	18–40			
Height, cm	>145	≥150		
BMI, kg/m^2^				
<2 wk postpartum	≥18.5 to ≤30.0	_	_	≥18.5 to ≤30.0
Prepregnancy		≥18.5 to ≤30.0	≥18.5 to ≤30.0	
MUAC, cm	≥23 to ≤ 33	_	_	≥23 to ≤33
Hemoglobin,^1^ g/L	>100	>100	No reported anemia	>100
Maternal health	No relevant past or current medical problems (e.g. gestational diabetes, pre-eclampsia)			
Smoking	No	No	No	No
Alcohol intake, mL	<50	<40	<50	<50
Diet^2^	Nonvegan or macrobiotic diet			
	≥15 g of ≥15 food groups each/d	≥15 g of 4 of 8 food groups/d	≥15 g of ≥15 food groups each/d	
MMN use				
3rd trimester	Iron and folic acid			
			Vitamin D and calcium	
lactation	Iron and folic acid			
			Vitamin D and calcium	
Fortified foods	Low habitual intake of highly fortified foods, expect iodized salt			
Delivery	Singleton			
Weeks of gestation	37–42			
Breastfeeding				
≤3.5 mo	Exclusive breastfeeding			
≤8.5 mo	Partially breastfeeding			
Infant inclusion criteria				
Birthweight, g	2500–4200			
Infant health	No congenital malformations that interfere with feeding or growth and development			
Visit 2 exclusion criteria	Cessation of, or nonexclusive, breastfeeding			
	Serious maternal illness			
	Infant length-, weight-for-age, or weight-for-length <-2 Z			
	Maternal MMN consumption during lactation other than stated above			
Visits 3 and 4 exclusion criteria	Cessation of breastfeeding			
	Infant length-, weight-for-age, or weight-for-length <-2 Z			
	Abnormal infant development based on Ages & Stages Questionnaire (abnormal performance on ≥2 of the 7 domains)			

1 Hemoglobin determined during the third trimester, or by maternal questionnaire in Denmark.

2 Using a locally appropriate and validated FFQ.

MMN, multiple micronutrients; MUAC, midupper arm circumference.

### Data and sample collection

**FIGURE 1:**
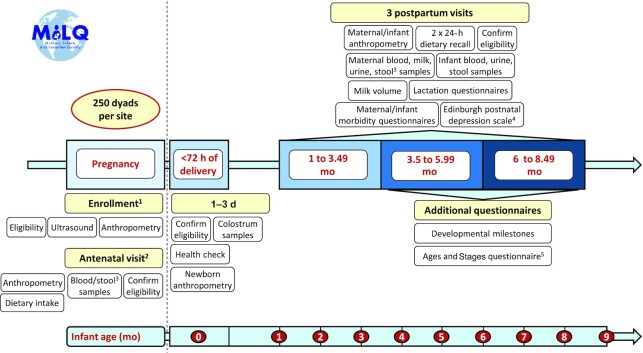
Mothers, Infants, and Lactation Quality Study timeline, questionnaires, and data and sample collection form schedule. ^1^3rd trimester. ^2^Late 3rd trimester, not done in Bangladesh. ^3^Maternal stool samples are not collected in Bangladesh. ^4^Not collected in Denmark. ^5^Only at visit 4 in The Gambia.

**FIGURE 2:**
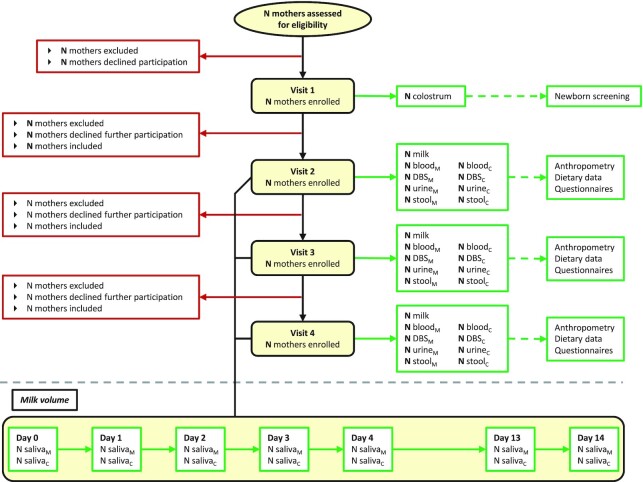
Mothers, Infants, and Lactation Quality Study participant flow chart, for all 4 study sites. ^1^Denmark will use 24-h test weighing, instead of the Dose-to Mother deuterium method and saliva collections, for milk volume measurements. _C_, child; DBS, dried blood spots; _M_, mother.

Data and biological samples are collected during recruitment (28 weeks of gestation), at 1–3 d postpartum (within 72 h of delivery), and at scheduled visits 2, 3, and 4 (1.0–3.49 mo, 3.5–5.99 mo, 6.0–8.5 mo postpartum). Samples collected include human milk, and maternal and infant blood, urine, and stool. Additional data collected includes maternal and infant anthropometry, dietary intakes, and morbidity. A detailed timeline of data and sample collection is provided in **Figures 1** and **2**. To estimate milk volume, 3 sites (Bangladesh, Brazil, and The Gambia) are collecting saliva samples as described below using deuterated water. In Denmark, where this method is generally not acceptable, the mothers are instructed by the research team to test weigh the infant as described below.

#### Questionnaires

The field staff use standardized questionnaires designed for the MILQ Study. Participant's information, pregnancy screening, maternal dietary data, and socioeconomic data is retrieved during recruitment (at visit 2 in Denmark). Each of the following visits is equipped with their unique set of questionnaires (Figure 1).

#### Anthropometry

Anthropometry measures are especially important for excluding mothers who are under- or overweight and excluding malnourished infants with abnormal birth size and Z scores ≤2. The methods that are used to perform accurate, precise, and standardized anthropometric measurements are carried out by standard methods, with regularly validated equipment. The anthropometric measurements include:

- Mother: weight, height, midupper arm circumference (MUAC) during pregnancy; (Denmark only at visit 2). Body composition using bioimpedance analysis (BIA) in Denmark and Brazil. BMI is calculated.
- Infant: weight, length, knee-heel length (in Denmark and The Gambia), MUAC, head circumference. Body composition is estimated from birth weight and length reported in health records, and by bioimpedance at visits 2, 3, and 4, in Brazil and Denmark, and by air displacement plethysmography until the infant reaches 8 kg,  (PEAPOD,  COSMED) in The Gambia. Z scores are calculated by the database system using WHO, 2006 growth standards

#### Milestones

Developmental milestones are evaluated at visits 3 and 4 to identify any infants with poor development, and to support the evidence that the milk MN concentrations are adequate for normal development. The final assessments at visit 4 includes Milestones (also collected at visit 3) and the Ages and Stages Questionnaires (19) (also administered at visits 2 and 3 in Brazil). Failure to meet normal developmental milestones in ≥2 domains will be considered abnormal during data analysis.

#### Dietary data

Dietary assessment of the mother is made by 2, 24-h recalls per period (visits 2, 3, and 4), and during pregnancy in Brazil and Denmark. One is taken during the study visits and the other during the week (≤10 d) before or after the study visit but not on an adjacent day. Data is collected about foods consumed the previous day based on a locally validated method. All sites have locally established procedures for food and nutrient intake assessment. Ideally, the total 6 d of recall data should reflect typical days of food intake, which may include weekend day(s). Data on the nutrient intakes of the mother enables classification of each woman based on the percent inadequate intakes (i.e. intakes below the Estimated Adequate Requirement) for each MN (calculated with the Intake Modeling, Assessment and Planning Program [IMAPP] software program), comparison between maternal intake and milk concentrations of MNs and other constituents, and comparison across sites.

### Specimen collection

All sample collection procedures have been developed with all field sites and collaborators at the analytical sites to ensure adequate sample collection (procedures and materials needed) for all planned analyses and are described in detail in the internal MILQ Study's “Manual of Operations and Procedures.” This detailed document has been shared with all field sites as the sample collection protocol, which included a completed sample collection form that states the specimen collected, subject ID, day and time, time of last meal, time of last feed, and volume and aliquots collected. All specimen collections are carried out under dim light or reduced light exposure. All samples are processed immediately and stored at –80°C until shipment to the analytical sites.

#### Colostrum

Colostrum is collected for antigen and MN analyses, with specifics to be determined based on volumes collected. At least 0.5 mL of colostrum is collected by manual expression from all women preferably 24–48 h after birth, but 48–72 h postpartum is acceptable. After cleaning the breast with mineral-free soap towelettes or deionized water, the sample is collected either into a collection tube or directly into a 2 mL amber storage tube. Samples collected by the mother at home are stored in the home freezer at −18°C until the first clinic visit.

#### Human milk and RNA

Milk samples at visits 2, 3, and 4 are collected preferably from the breast opposite from the one that the infant last fed from, using the Symphony electronic hospital-grade breast pump (Medela) for a full-breast expression. In the event of pump failure, milk is expressed using a hand pump or with manual expression, and noted on the collection form. The breast used for collection is cleaned with a mineral-free soap towelette or deionized water prior to milk expression. If ≥25mL of milk cannot be obtained from the first breast, the second breast is used to obtain a second full-breast expression, which will be noted on the collection form. A second milk sample from a subset of 50 women per study site is collected according to the protocol at visits 2, and 3, in Bangladesh, Brazil, and The Gambia, ∼2 wk after the respective main visit study, in order to estimate intraindividual variability in milk composition.

Milk RNA is collected in Denmark and The Gambia; 10 mL of the full-breast expression is used for RNA extraction. Under sterile conditions, the milk is centrifuged to obtain the RNA pellet, which is washed with PBS and stored under TRIzol in a DNA/RNA-free tube at –80°C. RNA is collected from 250 women at visit 2, and 50 women at visit 3 and visit 4, at both field sites.

#### Saliva and milk intake measurements

In the same periods that milk is collected, milk volume is measured in all women at each site, to determine how usual milk volume, which varies greatly among women, is related to nutrient concentrations and to enable estimation of total daily nutrient intake by the infant. Three of the field sites (Bangladesh, Brazil, and The Gambia) are using the International Atomic Energy Agency (IAEA) mother to child deuterium (D2M) method (https://www-pub.iaea.org/MTCD/Publications/PDF/Pub1429_web.pdf). Deuterated water (30 g) is administered to the mothers at visits 2, 3, and 4 and maternal and infant saliva samples collected at baseline, 1, 2, 3, 4, 13, and 14 d after dosing. Saliva samples are obtained from both mothers and infants, who are both weighed at baseline and day 14. Cotton balls are placed in the infant's mouth which are then squeezed by a syringe to retrieve the saliva. Denmark is estimating milk volume via 24-h test weighing of the infant before and after every feed for 24 h, plus 1 extra feed, to determine the 24-h intake. The infants are weighed with the same clothes and diaper at both weighings. Mothers are instructed by study personnel.

#### Blood

A venous blood sample is collected into an EDTA vacutainer from the mother on visits 2, 3, and 4. Fasting overnight or for 3–4 h prior to blood collection was ideal if possible. The mother's recall of the time of her last meal is recorded. A venous sample is collected from all infants at visit 2, and to reduce the number of infant blood draws to a total of 2, from 50% of infants at visit 3 and the other 50% at visit 4, randomly selected. In The Gambia, however, 100% of infants are sampled at each visit as well as the mother during pregnancy. The mother's recall of the time of the infant's last meal is also recorded.

Plasma is obtained by centrifugation of the blood sample at 1500 × *g* for 10 min at 4°C. The plasma is stored in aliquots in amber tubes. The buffy coat is collected into a DNAse/RNAse-free, sterile tube. The volume of the remaining RBCs is determined in order to wash the RBCs with an equal volume of 0.9% saline solution. The RBCs are centrifuged again and the supernatant is removed. This procedure is repeated until the supernatant is clear. After diluting with equal amounts of deionized water, the washed RBCs are aliquoted into amber tubes for storage. All aliquots are then frozen and stored at 70°C until analysis.

#### Dried blood spots

Dried blood spots (DBS) are obtained for iodine status assessment. Four (infant) or 6 (mother) spots are prepared on filter paper cards using 50 µL of whole blood from the EDTA vacutainer used for blood collection. If insufficient blood volume is obtained, 1 DBS is prepared. The blood is dispensed onto the filter paper without touching the paper, and after a drying period of 24 h at ≤25°C, the cards are stored in bags without the DBS touching each other. The DBS can be stored at –20°C until shipment.

#### Urine

Maternal and infant urine is collected to assess iodine status across sites, which will likely vary depending on consumption of locally fortified foods, e.g. iodized salt. Population daily iodine intake will be estimated from spot urinary iodine and creatinine concentrations. A midstream clean catch specimen is collected from the mother at the first voiding of the bladder during each visit. Infant urine collection is enabled using a cotton ball, pads, or urine collection bags that are placed inside a disposable diaper. The wet but not soiled (with feces) cotton ball or pad is removed from the diaper and the urine is obtained by squeezing the cotton balls in a syringe, or by squeezing the pad in a plastic bag with a missing corner. The urine is collected into a beaker and 2 aliquots from both the mother and infant urine are stored for iodine assessment.

#### Stool

Infant feces are used for microbiome profiling. Once the infant has a bowel movement, the stool is scored by the mother for consistency, color, and volume. About 1 g of the specimen is collected with the spoon (attached to the cap) of the feces collection tube. The spoon is placed into the collection tube and stored at –80°C. If the specimen is collected outside the clinic (not applicable in The Gambia, all stool sample collection here is conducted at the study site), it must be stored in the home freezer and delivered frozen, on ice, for –80°C storage as soon as possible. All participants have been instructed by the field study staff and have confirmed the availability of a freezer for eligibility of at-home stool collection. All times of collection and storage are recorded.

### Laboratory analyses

All samples except those to be used for iodine and milk volume determination are shipped to the USDA, Agricultural Research Service-Western Human Nutrition Research Center (USDA, ARS-WHNRC), Davis, CA, USA. At each site, the research personnel prepare the aliquots for the following analyses (Table 3). Samples for human milk vitamin D analysis will be sent from the USDA/ARS-WHNRC to the Nutritional Biomarker Laboratory, MRC Epidemiology Unit, University of Cambridge, Cambridge, UK.

#### Data management

A dedicated, REDCap database was developed at a single site (The Gambia) to be used across all study settings. Unique α-numeric study ID codes are generated and given to each participant. These will link all data collected from a specific individual. ID codes include a check letter to minimize ID errors. Data collection is carried out electronically, or on paper forms when necessary, by study staff. All forms in the field were designed specifically for the MILQ Study. Research assistants and supervisors review data on a daily basis before data from each field site is securely integrated into The Gambia REDCap database by a data manager. A copy of the final data obtained from the laboratory/analytical sites is reviewed by the study statistician before being securely integrated in a WHNRC-housed REDCap database. Both databases will be merged at the end of the field site data collection and integration. Data management and security procedures, including assurance of confidentiality, adhere to the Collaborative Institutional Training Initiative (CITI) and the Canadian Tri-Council Policy Statement on Ethical Conduct for Research Involving Humans (TCPS2 CORE) guidelines, and are outlined in full in the protocol at clinicaltrials.gov (NCT03254329, 18 August, 2017).

### Statistical analysis

#### Sample size calculations

Since the primary outcome is estimated key centiles of the distributions of nutrient concentrations in human milk, and centiles are invariant to monotonic transformation, sample sizes for constructing RVs are based on: *1*) estimating the 50th and 5th percentiles of each breast milk nutrient across time periods within each site and *2*) being able to establish equivalency among study sites before pooling data.

#### Estimating centiles

We aim for a monotonic transformation for each variable across all study sites to attain normal (Gaussian) distribution, from which parametric estimates of the centiles will be constructed. Regression models will be used to estimate the parameters of the distribution at each time period, but since such models will not be determined until the data are available, the sample size is based on a generic estimate for the p^th^ centile:

(1) \begin{eqnarray*} \bar{X} + {Z_p} \times s \times C{F_s} \end{eqnarray*}

where:

$\bar{X}$ = sample mean (or estimated predicted value from the regression model)

*Z_p_* = p^th^ centile of the standard normal distribution (negative for *P* <0.50)

$s$ = sample SD (or estimated SD from regression model)

*CF_s_* = correction factor for bias of s as an estimator of population SD (negligible for *n* >50).

The SE of this estimate, if *n* is >50, is approximately:

(2) \begin{eqnarray*} \frac{\sigma }{{\sqrt n }}\sqrt {1 + 0.5 \times Z_\alpha ^2} \end{eqnarray*}

The desired width of the CI is based on the distance of the 50th and 5th centiles from neighboring centiles, which yields a sample size invariant to the location, scale, or shape of the underlying distribution, as long as the variable can be normalized. This requires an overall sample size of 255 per time point to avoid overlap between the 95% CIs around the 5th and 10th centiles, and to estimate the sample median within 5 centiles with 95% confidence (**Table 4**). Once the centiles of interest are estimated for the transformed variable, they will be back-transformed to be expressed in the original units.

**TABLE 4:** Planned analyses in the Mothers, Infant, and Lactation Quality (MILQ) Study

Analyte Category	Analytes	Milk	Blood	Urine	Stool	Method	Ref.^1^
Fat-soluble	Vitamin A	X	X^P^			HPLC-MWL	(31)
vitamins and carotenoids	Vitamin E						
	α-carotene						
	β-carotene						
	β-cryptoxanthin						
	Lycopene						
	Lutein/zeaxanthin						
	Ergocalciferol (D_2_)	X	X^P^			UPLC-MS/MS	
	Cholecalciferol (D_3_)					CPBA	(32)
	25-OH-D_2_						
	25-OH-D_3_						
Water-soluble vitamins	Thiamin (B-1)	X	X^R^			HPLC-FLD	(33)
	Thiamin monophosphate (B-1)						
	Thiamin diphosphate (B-1)						
	Riboflavin (B-2)	X	X^P,R^			UPLC-MS/MS	(27)
	FAD (B-2)						
	FMN (B2)						
	Nicotinamide (B3)						
	Nicotinic acid (B-3)						
	Nicotinamide mononucleotide (B-3)						
	NAD (B-3)						
	NAD(P) (B-3)						
	Nicotinamide riboside (B-3)						
	Tryptophan (amino acid, B-3-related)						
	Pantothenic acid (B-5)						
	Pyridoxal (B-6)						
	Pyridoxine (B-6)						
	Pyridoxamine (B-6)						
	Pyridoxal 5-phosphate (B-6)						
	Biotin (B-7)						
	Folic acid (B-9)						
	5-methyl tetrahydrofolate (B-9)					CPBA^2^	(34)
	Cobalamin (B-12)	X	X^P^			CPBA	(28, 35)
Choline and related	Choline	X	X^P^			UPLC-MS/MS	(36)
metabolites	Phosphocholine						
	Glycerophospho choline						
	Betaine						
	Carnitine						
	Creatinine						
	Dimethylglycine						
	Methionine						
	Trimethylamine N-oxide						
B-12 biomarkers	Methylmalonic acid		X^P^			UPLC-MS/MS	(37)
	Homocysteine		X^P^			HPLC-FLD	(38)
Minerals and	Iron	X	X^P^			ICP-MS	(39)
trace elements	Copper						
	Zinc						
	Selenium						
	Sodium						
	Potassium						
	Magnesium						
	Calcium						
Iodine status	Iodine	X		X		ICP-MS	(40)
	Thyroglobulin		X^DBS^			ELISA	(41)
	Thyroid-stimulated hormone						
	Total thyroxine						
Macronutrients	Protein	X				NIR-spectroscopy	(42)
	Fat						
	Carbohydrates						
Glycomics	Human milk oligosaccharides^3^	X				HPLC Chip/TOF-MS	(43)
	Human milk proteomics^4^	X				UPLC-QqQ-MS	(44)
Metabolomics	Biocrates MxP® QUANT 500^3^	X	X^P^			UPLC-MS/MS	(45)
Inflammation markers	α-1-acid glycoprotein (AGP)		X^P^			CPBA	(46)
	C-reactive protein (CRP)						(47)
	IL-1β, IL-4, IL-8, IL-6, IL-10, IL-33, TNF-α, IFN-γ	X	X			MSD immunoassay plates	(48)
Iron status	Soluble transferrin receptors		X^P^			CPBA	(49)
	Ferritin						(50)
Hormones	Leptin, insulin, and adiponectin	X	X			MSD immunoassay plates	(48)
Genetics	Single nucleotide polymorphism		X^B^			GWAS	(51)
	CpG methylation patterns		X^B^			EWAS	(52)
Microbiome	Microbial community				X	16s RNA sequencing	(53)
					X	Shotgun metagenomics	(54)
Transcriptomics	mRNA transcriptome	X^RNA^				RNA seq	(55, 56)

Analyzed in: ^B^buffy coat, ^DBS^dried blood spots, ^P^plasma, ^R^RBCs, ^RNA^RNA.

1 References that describe methods used for the analysis or on which the nonpublished methods are based on: CPBA, competitive protein binding assay; EWAS, epigenome-wide association; FLD, fluorescence detection; GWAS, genome-wide associations; ICP, inductively coupled plasma MS/MS; MSD, Meso Scale Discovery; MWL, multi-wavelength detection; NIR, near infra red; QqQ-MS, triple quad MS; TOF, time of flight; UPLC, ultra-performance-LC.

2 Plasma folate is analyzed by CPBA.

3 https://lebrilla.faculty.ucdavis.edu/research/nutritional-glycomics/.

4 https://biocrates.com/mxp-quant-500-kit/.

For maternal and infant blood variables, it is likely that blood samples will not be available from all participants, so the sample size will be reduced by necessity. However, if it is determined that it's acceptable to pool information from ≥2 study sites to construct RVs for infant blood variables, the precision of the estimate will be acceptable.

#### Establishing equivalency among study sites

For each nutrient and time period, pairwise comparisons using the 2, 1-sided test (TOST) method will be used to compare the means of the 4 sites for equivalence (20). If differences >0.3 SD are detected, a follow-up test will be conducted to compare each mean to the combined means of the other 3 sites, to determine which sites can be pooled. A sample size of 200 per site in each time period is sufficient to detect differences of 0.25 SD with 80% power or differences of 0.30 SD with 90% power. Therefore, the planned sample size of 250 per site and time period will provide adequate power to determine whether 1 site is different from the others (**Table 5**).

**TABLE 5:** Required sample sizes for testing equivalence

Differences (site means)	Sample size/site 80% power	Sample size/site 80% power
0.20 SD	310	429
0.25 SD	199	275
0.30 SD	139	191

#### Sample size estimates for other analyses

For some secondary outcome variables, e.g. free amino acids (FAA) in infant plasma, a complete sample set for analyses is not feasible or available. In such cases, only 100 samples for each site and time point are analyzed, assuming a priori that the values are similar enough to pool between ≥2 of the sites. If this is not possible, within-site percentiles will be presented with a caveat that these should not be considered as RVs, as precision is limited. However, 100 per time point is ample for looking at relations between secondary outcome variables and other outcomes, such as growth. Nevertheless, banked samples are available if initial data analyses show that analysis of >100 samples per point is needed.

Future exploratory analyses will also assess the relations between milk nutrient concentrations and other collected information, including milk volume, child growth, child developmental milestones, and maternal and infant plasma values. A sample size of 140 paired data points per site and time period is adequate to detect a correlation between continuous variables of 0.30 or higher with 95% power within each site, assuming a 2-sided alternative hypothesis, and therefore the planned sample size of 250 per site and time point is more than sufficient for these analyses.

#### Data analysis plan

**FIGURE 3:**
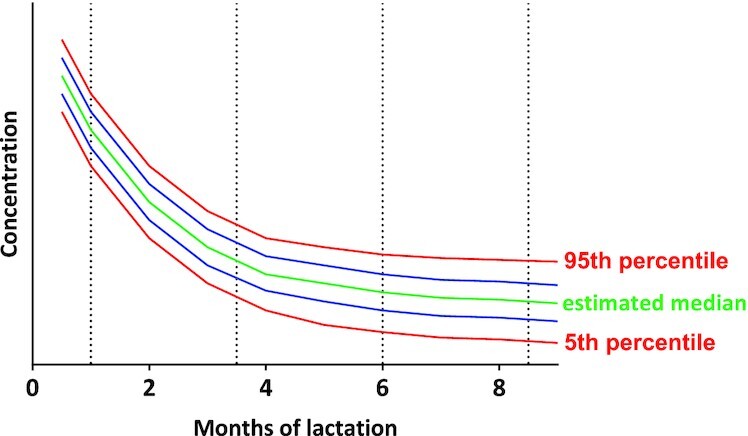
Hypothetical graphical illustration of reference values that may result for a micronutrient.

The measured human milk nutrient concentrations and milk volume will be used to estimate daily nutrient intakes of the infants. The RVs will be based on these infant nutrient intakes, and developed and expressed as percentiles in these well-nourished, but nonsupplemented, population groups, following the principles used by the Intergrowth-21st Project (21), which are based on methods developed in the construction of the WHO Child Growth Standards (22) (illustrated in **Figure 3**). Criteria will be created for normal growth and development, and adequate nutritional status for each nutrient in question. Data from children who do not meet these criteria will be removed from the construction of the RVs.

For each nutrient and time period, the distribution of the human milk variable will be examined and extreme outliers will be investigated and, if needed, removed. Box–Cox and other transformations will be used to normalize each variable, preferably using the same transformation across study sites, and the Shapiro–Wilk statistic to assess normality; skewness, kurtosis. The general shape of the distribution will also be examined. Parameters will be compared among study sites as described above, and information from the sites will be combined if the sites are deemed to be equivalent, or if differences have no material effect on key percentiles; otherwise, different sets of RV centiles may be constructed per site, or with only 2 or 3 pooled sites.

Parameters from the identified distribution will be estimated from the data, and centiles of interest (5th, 25th, 50th, 75th, 95th) will be estimated for each nutrient based on the theoretical percentiles of the underlying distribution, as described above. Smoothing techniques such as cubic splines will be employed, assuming the centiles follow smooth and continuous functions. Tables and curves of estimated centiles will be created for each nutrient and time period. SAS for Windows Release 9.4 (SAS Institute) will be used for all analyses.

### Additional study outcomes

Besides the primary study outcome, the construction of the RVs, secondary study outcomes are included in the study design and categorized into outcomes that: *1*) will be used for the construction of the RVs, *2*) will not be used to construct RVs, and *3*) country-specific measurements that are not used for RV development. These outcomes are summarized by category in **Table 6**.

**TABLE 6:** Secondary outcomes of the Mothers, Infants, and Lactation Quality (MILQ) Study

Outcome category^1^	Bangladesh	Brazil	Denmark	The Gambia
Used for	Macronutrients, human milk oligosaccharides, peptides, and proteins, metabolomics			
constructing RVs	Micronutrient status of mothers and infants assessed in blood samples			
	Milk volume by D_2_O		24-h infant test weighing	Milk volume by D_2_O
	Iodine and maternal and infant iodine status biomarker			
Not used for	Maternal and infant dietary intakes			
constructing RVs	Maternal and infant anthropometry			
		Maternal body composition (bioimpedance)		
		Infant body composition (bioimpedance)		Infant body composition
				(air displacement plethysmography)
	Maternal and infant morbidity			
	Infant motor development assessment (3.5–5.9 and 6–8.5 mo pp)^2^			
	Infant development using the Ages and Stages questionnaire (6–8.5 mo pp)			
	Infant microbiome			
	Maternal inflammatory markers (28–30 weeks of gestation)			
Country-specific outcomes		Maternal nutrient intake at 35–37 weeks of gestation		
		Maternal microbiome^3^		Maternal microbiome^3^
			Maternal hemoglobin A1c^4^	
			Maternal markers of insulin sensitivity^5^	
			Maternal lipid panel^6^	

1 Unless otherwise stated, the time periods for the secondary outcome and analysis objectives are identical to those for the primary outcome (1–3.4, 3.5–5.9, 6–8.5 mo postpartum).

2 Based on WHO's motor milestones.

3 28–40 weeks of gestation, and 1 to 8.5 mo pp.

4 28–30 weeks of gestation, measured in whole blood.

5 28–30 weeks of gestation: insulin, C-peptide, and leptin (plasma).

6 28–30 weeks of gestation: total cholesterol, HDL-cholesterol, LDL-cholesterol, very LDL-cholesterol, and triglycerides (plasma). D_2_O, deuterated water; pp, postpartum, RV, reference value.

### Ethics, dissemination, and trial status

Full ethical approvals at all study sites were obtained from:

- The Institutional Review Board of the University of California, Davis, CA, USA (IRB ID: 920618–1, Protocol HRP-503-MILQ IRB, Department of Health and Human Services FWA No: 00004557).
- The Internal Review Boards of the International Centre for Diarrhoeal Disease Research, Bangladesh (icddr, b; PR-17085).
- The National Commission for Research Ethics (2.086.708, 2.875.218, 4.865.685), the Research Ethics Committees of the Municipal Secretariat of Health and Civil Defense of the State of Rio de Janeiro and of the Maternity School of Rio de Janeiro Federal University, (1.948.992, 2.769.611, 4.449.007); and the Municipal Secretary of Health and Civil Defense of the State of Rio de Janeiro (2.100.255), Brazil; Project number: 64767717.4.0000.5275.
- The Committees on Biomedical Research Ethics for the Capital Region of Denmark (H-17015174).
- The joint Gambia Government/MRC The Gambia Ethics Committee (SCC 1572v1.1, Project ID/ethics ref: 22768).

The MILQ Study was registered at clinicaltrials.gov as NCT03254329 (18 August, 2017). Study progress was discussed among all field sites and the USDA/ARS-WHNRC, the main analytical site, and location of the Principal Investigator (PI), in biweekly conference calls. Updates of the study are provided monthly in conference calls between the PI and the funding agency, which are reported back to the field sites. Results are presented at national and international nutrition-related conferences, and in peer-reviewed journals. MILQ meetings will be hosted at 1 study site each year. Funding has been secured for students and researchers to visit other study sites for professional development. Deidentified data will be available in a public repository after full publication of the primary study outcomes (RVs) for milk MNs and country-specific approvals. The recruitment phase started between September 2017 (Denmark) and May 2018 (The Gambia) (**Table 7**). The analytical work began in May 2019 and is expected to be completed by December 2022.

**TABLE 7:** Trial status

	Bangladesh	Brazil	Denmark	The Gambia	USA^1^
Recruitment start	04/2018	01/2018	09/2017	05/2018	—
Completion of field site work	Expected by 03/2022	Expected by 03/2022	12/2019	Expected by 03/2022	—
Sample analyses start	—	—	—	—	05/2019
Completion of sample analyses	—	—	—	—	Expected by 12/2022

1 Additional analytical sites are Bengaluru, India (milk volume); Cambridge, UK (vitamin D in human milk); Zürich, Switzerland (iodine and iodine status in milk, dried blood spots, and urine).

## Discussion

Although the importance of human milk in maternal and infant nutrition has long been recognized, the MN intake recommendations for breastfeeding infants and lactating women are still based on old, uncertain, or even incorrect data (4) and are established mainly as Adequate Intakes for infants due to the lack of experimentally determined estimates of milk composition and nutrient requirements (23). Further, the dynamic changes in concentrations of many milk nutrients across lactation are not reflected in the current recommendations, which can lead to erroneous status evaluations (24, 25). Therefore, given the importance of adequate nutrient intake during the first 1000 d of life (26), which includes the WHO-recommended 6-mo period of EBF (1), this systematic, longitudinal assessment of the nutrient composition of human milk, in particular for MNs, using validated methodologies, is indispensable for correctly evaluating nutrient intake adequacy of breastfeeding infants.

We have developed and validated multiple novel methods for collecting and analyzing human milk (24, 27–30), which will be used or further optimized for the nutrient assessment in this study. The identical collection protocols and methods for analyzing human milk and blood samples minimize method bias and enable direct comparison between field sites. The inclusion and exclusion criteria, and collection of information on diets, anthropometry, and infant developmental milestones provide supportive information that participants are healthy and nutrient sufficient.

The 4 field sites are different in demographics, socioeconomic status, diets, and cultural practices which may preclude combining data across sites to establish the RVs, but the required number of participants per site allows for the creation of study-site-specific RVs if necessary.

The longitudinal data obtained by the staggered sample collection protocol will allow for the development of new dynamic RVs, providing percentile ranges for every stage of lactation covered in this study. These carefully developed RVs, in conjunction with the milk volume data, will be available for re-evaluating intake recommendations for infants, young children, and lactating women, and as benchmarks against which to evaluate human milk quality, and the effects of future nutrient intervention trials on milk composition.

## Notes

This work was supported by the Bill & Melinda Gates Foundation grant numbers OPP1148405 and INV-002300, and intramural USDA-Agricultural Research Service project 5306-51000-004-00D. Additional funding support for Brazil: GK (CNPq, 309433/2017-7 and Faperj, E-26/202.907/2017) and DBM (Faperj, E-26/201.904/2020 and E-26/201.903/2020). Additional funding for Gambia for SEM and AMD (UK Medical Research Council, MR/P012019/1) and SEM (Wellcome Trust fund, 220225/Z/20/Z). SHC has received funds from the University of Copenhagen. USDA is an equal opportunity employer and provider. The funder will have no involvement in the study design, collection, management, analysis, and interpretation of data, writing of the report, or the decision to submit the report for publication.

Author disclosures: The authors report no conflicts of interest.

Abbreviations used: DBS, dried blood spots; EBF, exclusive breastfeeding; MILQ Study, Mothers, Infants, and Lactation Quality Study; MN, micronutrient; MUAC, midupper arm circumference; RV, reference value.
